# Minimally Invasive Pancreatoduodenectomy for Pancreatic Cancer: Current Perspectives and Future Directions

**DOI:** 10.3390/cancers18020197

**Published:** 2026-01-07

**Authors:** Munseok Choi, Chang Moo Kang

**Affiliations:** 1 Department of Surgery, Yongin Severance Hospital, Yonsei University College of Medicine, Yongin-si 16995, Republic of Korea; cms2598@yuhs.ac; 2 Division of HBP Surgery, Department of Surgery, Yonsei University College of Medicine, Seoul 03722, Republic of Korea; 3 Pancreatobiliary Cancer Center, Yonsei Cancer Center, Severance Hospital, Seoul 03722, Republic of Korea

**Keywords:** minimally invasive surgery, pancreatoduodenectomy, pancreatic cancer, laparoscopy, robotic surgery

## Abstract

**Background:** Minimally invasive pancreatoduodenectomy (MIPD) has evolved from an experimental technique to a feasible surgical option for pancreatic cancer in selected settings. However, its oncologic adequacy, safety, and generalizability remain debated, particularly given the biological aggressiveness of pancreatic ductal adenocarcinoma (PDAC) and the technical complexity of the procedure. **Methods:** This narrative review critically summarizes contemporary evidence regarding MIPD for pancreatic cancer, with particular attention to randomized controlled trials (RCTs), meta-analyses, and large observational studies. We distinguish findings derived from mixed periampullary tumor cohorts from those specific to PDAC and evaluate methodological limitations, learning-curve effects, and sources of heterogeneity across studies. **Results:** Recent RCTs and meta-analyses demonstrate that, when performed by experienced surgeons in high-volume centers, MIPD achieves perioperative outcomes comparable to open pancreatoduodenectomy, with advantages including reduced blood loss, shorter hospital stay, and faster functional recovery. Importantly, oncologic parameters such as R0 resection rates and lymph node yield appear equivalent between approaches, although robust long-term survival data from PDAC-specific RCTs remain lacking. Emerging evidence supports the feasibility of MIPD in complex clinical scenarios, including after neoadjuvant therapy, in frail or elderly patients, and in selected cases requiring vascular resection. Nonetheless, outcomes are strongly influenced by surgeon experience, institutional volume, and patient selection. Cost-effectiveness analyses and data from lower-volume centers remain limited. **Conclusions:** Current evidence supports MIPD as a viable alternative to open surgery for pancreatic cancer in carefully selected patients treated at specialized centers. However, claims of oncologic superiority are premature. Future research should focus on PDAC-specific randomized trials, standardized quality metrics, and strategies to mitigate learning-curve and resource-related barriers to broader implementation.

## Simple Summary

Minimally invasive pancreatoduodenectomy has emerged as a feasible option for pancreatic cancer in expert centers. Current evidence shows comparable safety and oncologic adequacy to open surgery in selected patients, while long-term PDAC-specific data and standardization remain needed.

## 1. Introduction

Pancreatic cancer, largely pancreatic ductal adenocarcinoma (PDAC), is among the most aggressive malignancies with rising incidence and mortality. It currently ranks as the fourth leading cause of cancer-related death and is projected to become the second by 2030 [1]. Surgical resection offers the only chance for cure, typically via pancreatoduodenectomy (PD) for tumors of the pancreatic head and periampullary region. However, PD is an extensive operation with historically high risks; early reports documented perioperative mortality rates near 45%, though advances in surgical technique and perioperative care have reduced mortality to 2% in high-volume centers [2,3]. Minimally invasive pancreatoduodenectomy (MIPD), via laparoscopic or robotic approaches, has emerged over the past two decades as a potential alternative to open surgery (OPD) [4,5,6]. While minimally invasive distal pancreatectomy is now standard for left-sided tumors, adoption of MIPD has been slower due to technical complexity and concerns about oncologic adequacy [7]. Notably, PDAC presents unique challenges for MIPD compared to other periampullary tumors because PDAC often involves local vascular and neural invasion and extensive lymph node spread [8]. Thus, much of the initial evidence for MIPD comes from mixed periampullary tumor series, and its applicability specifically to PDAC remains debated [9,10,11].

This narrative review provides a structured appraisal of the current evidence on MIPD for pancreatic cancer. We outline the advancements in technical feasibility and training that have enabled MIPD and provide a critical review of evidence from randomized controlled trials and meta-analyses. Furthermore, we discuss oncologic outcomes specifically in the context of PDAC and examine key factors such as the learning curve, cost-effectiveness, and specialized patient populations, including those requiring vascular resection, receiving neoadjuvant therapy, or presenting with significant frailty. We then consider future perspectives for the expanding role of MIPD. The goal is to objectively assess whether and in what contexts MIPD can match or exceed open surgery, acknowledging limitations of current data and avoiding overstatement of benefits. All sections emphasize critical evaluation of study design, biases, and generalizability to guide an evidence-based understanding appropriate for a high-impact surgical oncology audience.

## 2. Technical Feasibility of MIPD

Early attempts at laparoscopic pancreatoduodenectomy (LPD) in the 1990s were isolated successes, but the procedure remained experimental for years [12]. In recent years, substantial improvements in surgical technology and technique have enhanced the feasibility of MIPD. Advanced laparoscopic instrumentation, high-definition 3D visualization, and energy devices have enabled refined dissection and hemostasis even in the deep retroperitoneal plane. The advent of robotic surgical platforms has further expanded technical capabilities, offering magnified three-dimensional optics, wristed instruments with greater articulation, tremor filtration, and motion scaling [13,14,15]. These features facilitate precise dissection around critical vasculature and complex reconstruction, making robotic PD (RPD) an increasingly popular approach in experienced centers. Standardization of the operative steps for both LPD and RPD has been proposed to ensure safety and reproducibility. For example, stepwise approaches to LPD and formalized robotic techniques (such as a 17-step RPD technique) have been described to streamline the operation [13,14,16].

Despite these advances, MIPD remains technically demanding with a steep learning curve. Multiple studies indicate that surgeons must perform a substantial number of cases to achieve proficiency [17,18,19]. A systematic review of learning curves estimated that approximately 35 cases are required to surmount the learning curve for LPD (median 34.1 cases, 95% CI 30.7–37.7) and a similar volume (36.7 cases, 95% CI 32.9–41.0) for RPD; notably, these figures come from high-volume centers and may not generalize to all practice settings [19]. This high learning threshold reflects the complexity of vascular dissection, pancreatico-enteric anastomosis, and complication management in minimally invasive settings. In the early learning phase, MIPD is associated with longer operative times and potentially higher risks, underscoring the need for structured training and proctorship [20,21]. Mitigation strategies have been proposed to help surgeons and centers climb the learning curve safely. These include intensive training programs with stepwise skill acquisition, utilization of simulation (virtual reality modules, procedure rehearsals) and animal models for pancreaticoenteric anastomosis practice, video coaching, and visiting mentorship from high-volume centers [22,23,24,25,26]. Case selection algorithms are of paramount importance; during the early stages of a surgeon’s MIPD experience, prioritizing lower-risk cases—such as those involving smaller tumors without vascular involvement or a favorable soft pancreatic texture—can significantly improve clinical outcomes until greater proficiency is achieved.

The impact of the learning curve is evident even in clinical trials: the Dutch LEOPARD-2 RCT had to be halted due to an unexpected excess of mortality in the laparoscopic arm, attributed in part to the participating centers’ limited prior MIPD experience [27]. Low-volume centers may particularly struggle to accumulate the case numbers needed for proficiency, raising concern about generalizability of excellent outcomes achieved in expert centers. This challenge has spurred calls for centralization of MIPD in high-volume institutions and the development of credentialing standards or minimum case volumes for surgeons embarking on MIPD programs.

Overall, while technical feasibility of MIPD is now well-established, it hinges on significant expertise and institutional support. Centers that have invested in dedicated training and adopted the robotic platform report that MIPD can be performed as safely and effectively as open surgery [28]. For widespread adoption, dissemination of standardized techniques, formal training pathways, and mitigation of the learning curve are critical to ensure MIPD’s technical success and patient safety.

## 3. Evidence from RCTs and Meta-Analyses of MIPD vs. OPD

High-level evidence comparing minimally invasive to open PD has begun to accumulate, though it is important to distinguish the scope of each. Table 1 summarizes the major randomized trials to date comparing MIPD with OPD, including key endpoints and outcomes.

These RCTs collectively suggest that MIPD can achieve at least equivalent short-term outcomes to open surgery in high-volume, expert settings, with some trials showing specific advantages of MIPD. Laparoscopic trials conducted in India, Spain, the Netherlands, China, and Korea consistently demonstrated no compromise in oncologic endpoints, such as resection margins and lymph node harvest, and showed no increase in major morbidity or mortality compared to open surgery [27,29,30,31,32]. Several demonstrated statistically significant benefits of MIPD: notably shorter hospital stays, reduced intraoperative blood loss, and in one trial, PADULAP trial, a lower rate of severe complications and better overall recovery profile with LPD [30]. It must be emphasized, however, that these advantages were shown in selected patient populations and by surgeons already adept at MIPD. The patient inclusion criteria often encompassed all periampullary tumors. This means a sizable fraction of patients in each trial did not have true pancreatic adenocarcinoma—for example, only 27.2% of patients in the K-MIPS trial had PDAC [32]. Because ampullary and bile duct tumors typically offer a better prognosis and lower technical complexity—characterized by a softer pancreas and reduced inflammatory response compared to PDAC—the trial results might provide an overly optimistic view that does not entirely represent the challenges of treating PDAC. Nonetheless, the trials establish that in experienced centers, MIPD is not inferior to open surgery on key metrics and can accelerate recovery [31,32].

It is also notable that LEOPARD-2 stands out with a cautionary outcome: an unexpectedly high 90-day mortality in the laparoscopic arm led to early termination [27]. Although the mortality difference (LPD vs. OPD, 5 deaths vs. 1 death, *p* = 0.2) did not reach statistical significance due to sample size, it breached safety stopping rules. No specific technical failure was identified as the cause; rather, it highlighted that outcomes were worse during the early phase of adopting MIPD at multiple centers. This underscores that adequate MIPD experience is a precondition for matching open surgery outcomes, and RCTs must ensure participating surgeons have cleared their learning curves. Subsequent trials avoided this issue by involving high-volume surgeons or imposing training run-ins to ensure safety [31,32].

Several meta-analyses have synthesized the RCT data and large non-randomized series. Sattari et al. (2023) pooled results of early RCTs comparing LPD vs. OPD [35]. Consistent with individual trials, LPD was associated with a longer operative time by about 75 min, but showed advantages including 115 mL less blood loss, roughly one day shorter ICU stay, and reduced need for blood transfusion and surgical site infection rates. Importantly, no significant differences were found in oncologic outcomes: the number of lymph nodes retrieved and R0 resection rates were equivalent between LPD and OPD. This meta-analysis, covering patients mostly with periampullary cancers, supports the notion that MIPD can uphold oncologic standards while conferring modest perioperative benefits, provided the surgery is performed in appropriate settings. Another recent network meta-analysis (Joseph et al., 2024) incorporated newer trials to compare open, laparoscopic, and robotic approaches [36]. It concluded LPD shows the clearest benefit such as shorter hospital stay, less blood loss over open, whereas evidence for RPD’s advantage remains inconsistent. The heterogeneity in robotic outcomes across studies suggests that RPD’s learning curve and resource demands might have tempered its measured benefits, and that further large trials are needed to determine if RPD can outperform open surgery when performed widely.

In summary, current Level 1 evidence suggests that MIPD is a feasible and safe alternative to open PD, offering comparable oncologic outcomes coupled with certain short-term advantages in recovery, provided it is conducted within the context of significant surgical expertise. However, most trials included mixed tumor types, and their findings must be carefully interpreted for PDAC-specific practice. We next examine the evidence focusing specifically on oncologic outcomes in pancreatic cancer, where long-term survival and tumor clearance are paramount.

## 4. Oncologic Outcomes of MIPD Versus OPD in Pancreatic Cancer

Because PDAC has distinct biological aggressiveness and surgical challenges, it is crucial to ascertain whether MIPD can achieve oncologic outcomes including margin-negative resection, adequate lymphadenectomy, and overall survival on a par with open surgery for this specific disease [5,8]. Early fears were that the minimally invasive approach might compromise tumor clearance or regional lymph node dissection. Fortunately, growing evidence suggests that MIPD can meet the oncologic benchmarks of PDAC surgery, though claims of superiority should be viewed with caution pending long-term randomized data [37,38] (Table 2).

Pathologic radicality appears to be maintained with MIPD in PDAC. A 2019 meta-analysis by Jiang et al. focused specifically on PDAC (8 studies, >15,000 patients) comparing LPD vs. OPD [40]. The pooled results showed LPD achieved a higher R0 resection rate and harvested more lymph nodes on average than open surgery. Specifically, MIPD patients had slightly better odds of negative margins and a greater number of nodes examined, a finding likely reflecting the meticulous dissection and enhanced visualization in minimally invasive approaches. Other studies echo these findings: for example, a multi-center propensity-matched comparison found no difference in margin status between RPD and OPD, even in complex cases, and in some reports MIPD yielded more nodes than OPD [41,43]. The improved visualization and fine instrument control in MIPD are theorized to facilitate thorough lymphadenectomy [4,44]. It is worth noting, however, that while a higher lymph node count can improve staging accuracy, it has not yet been proven to translate into improved survival for MIPD patients—it simply indicates oncologic equivalence or potential technical advantage in node dissection. Similarly, claims that MIPD achieves higher R0 rates than open surgery may reflect patient selection, as surgeons might attempt MIPD in anatomically favorable tumors, or the expertise of high-volume centers rather than an intrinsic benefit of laparoscopy/robotics. The consensus from controlled studies is that R0 and lymph node yield are no worse with MIPD, and likely equivalent to open surgery. Thus, concerns about oncologic inadequacy of MIPD have not been borne out in aggregate data—a critical prerequisite if MIPD is to be considered for cancer care.

Regarding short-term cancer outcomes and adjuvant therapy, one hypothesized oncologic advantage of the faster recovery associated with MIPD is the earlier initiation of adjuvant chemotherapy for PDAC [45]. Delays or omission of adjuvant therapy negatively impact survival in PDAC. Some retrospective studies suggest MIPD patients are more likely to start adjuvant chemotherapy on time. For instance, Croome et al. compared LPD vs. OPD for PDAC and found a significantly lower proportion of LPD patients had >90-day delay or omission of adjuvant therapy (5% vs. 12% in OPD, *p* = 0.04) [11]. This corresponded with a longer progression-free survival in the MIPD group, hinting that quicker postoperative recovery might confer a real survival benefit. Another institutional series by Choi et al. reported improved disease-free survival (median 34 vs. 23 months, *p* < 0.05) for LPD over OPD in PDAC, even though overall survival was similar [9]. These findings are provocative but must be interpreted cautiously; as non-randomized studies, they could reflect selection bias, whereby surgeons may have offered MIPD to patients with smaller or biologically less aggressive tumors, or those in better overall condition. Moreover, a large database analysis by Nussbaum et al. found no significant improvement in adjuvant therapy use or timing with MIPD for PDAC, suggesting that in broader practice, the advantage may not consistently manifest [39]. The recently completed K-MIPS trial did note a shorter time to functional recovery and a trend toward earlier adjuvant therapy with LPD, but long-term follow-up is needed to see if this impacts survival [32]. At present, it would be overstating to claim MIPD improves overall survival in PDAC—there is no conclusive randomized evidence of that. What can be said is that MIPD facilitates timely postoperative recovery, which is a necessary step toward receiving adjuvant treatment, a known determinant of survival in PDAC.

Long-term survival outcomes after MIPD vs. OPD for PDAC are not yet defined by RCTs. Retrospective comparisons have generally found no significant difference in overall survival when adjusting for disease stage. For example, Choi et al. (2020) reported 5-year survival curves for PDAC that were nearly identical between LPD and OPD (median 45 months, *p* = 0.22) [9]. Similarly, the meta-analysis by Jiang et al. found equivalent 5-year overall survival between MIPD and OPD groups [40]. These data reassure that MIPD does not compromise the curative intent of surgery—patients are not dying sooner because of minimal invasiveness. However, demonstrating a survival benefit of MIPD is challenging, since surgical approach likely has a smaller impact than tumor biology and adjunct therapy on long-term outcomes [46,47]. Any slight improvements via earlier chemo or reduced complications would need very large samples to translate into detectable survival differences. A new Chinese RCT focusing specifically on PDAC examined short-term outcomes in 258 PDAC patients randomized to LPD vs. OPD [42]. It showed no difference in 30-day morbidity or mortality between approaches and confirmed the expected longer operative time and lower blood loss with LPD. While not powered for survival, it adds to evidence that in PDAC, MIPD can be considered non-inferior to open surgery in the perioperative period. Long-term follow-up from such trials, as well as ongoing studies, will be needed to see if survival curves diverge over years.

However, interpretation of MIPD oncologic outcomes is hampered by a lack of standardized reporting across studies, which leads to inconsistent definitions and incomplete data. For instance, margin status criteria vary, and many studies do not report achieving the recommended minimum of 12 lymph nodes [48,49]. Similarly, heterogeneity in reporting the extent of resection (e.g., inclusion of vascular resection) and neoadjuvant/adjuvant therapy further confounds comparisons [50]. Consequently, comparisons of MIPD versus open PD are prone to bias from such heterogeneity, making it difficult to discern MIPD’s true oncologic impact. Thus, establishing a PDAC-specific consensus on uniform definitions and comprehensive data reporting—covering margins, lymph node yield, extent of resection, and neoadjuvant/adjuvant therapy—is essential, as a prerequisite for unbiased comparisons between MIPD and open surgery.

In summary, current oncologic evidence in PDAC indicates that MIPD, when performed by skilled surgeons on appropriate tumors, achieves similar cancer clearance and survival outcomes as open PD. Some single-center experiences even suggest improved disease-free intervals or more prompt adjuvant therapy, but these findings require confirmation. Given the limitations of retrospective analyses, any implication that MIPD is oncologically superior to open surgery should be tempered. It would be more accurate to conclude that MIPD has demonstrated oncologic safety and equivalence for PDAC thus far. Ongoing trials and longer-term data will clarify whether any subtle oncologic advantages will translate into better survival, or whether MIPD’s main benefits will remain in the realm of perioperative quality of life rather than cancer-specific outcomes.

## 5. Special Populations and Expanded Indications

One striking development in recent years is the expansion of MIPD indications beyond the “ideal” straightforward cases [5,10]. As experience has grown, surgeons have cautiously pushed the envelope of MIPD to encompass scenarios traditionally deemed high-risk or contraindicated for laparoscopy/robotics. Here, we discuss how MIPD is being applied in special patient populations and challenging tumor scenarios—including those receiving neoadjuvant therapy, the elderly and frail, and tumors requiring vascular resection or other complex techniques. In each case, the evidence is largely from retrospective studies, so a critical eye on potential biases is needed.

### 5.1. MIPD After Neoadjuvant Therapy for Borderline/Locally Advanced PDAC

Neoadjuvant therapy (NAT), typically multi-agent chemotherapy with or without chemoradiation, is increasingly used for borderline resectable or locally advanced PDAC prior to surgery [51,52]. NAT can downstage tumors, sterilize micrometastases, and select out aggressive biology. However, NAT also induces inflammation and fibrosis around the pancreas, often blurring tissue planes and making dissection more challenging [53,54]. There were concerns that post-NAT fibrosis, especially around vessels like the superior mesenteric artery or portal vein, would make an already tough minimally invasive dissection even harder, possibly negating the feasibility of MIPD [55]. Indeed, a large meta-analysis reported that only approximately 17% of pancreatoduodenectomies following neoadjuvant therapy were performed using a robotic approach, reflecting cautious and selective adoption of minimally invasive techniques in this setting [56].

Recent studies are beginning to show that MIPD after neoadjuvant therapy might be feasible in experienced hands, with outcomes comparable to open surgery. Nassour et al. analyzed the U.S. National Cancer Database for PDAC patients who underwent NAT followed by surgery [55]. They found only 5% of these cases were done via RPD, reflecting rarity, but those RPD cases had encouraging outcomes: RPD was associated with a higher likelihood of an adequate lymph node harvest (≥15 nodes), a higher rate of receiving adjuvant chemotherapy, shorter hospital stays, and no difference in 90-day mortality compared to open. Long-term survival was equivalent between RPD and OPD after NAT (e.g., 5-year survival 22% in both, *p* = 0.879). This suggests that carefully selected patients can undergo MIPD after NAT without compromising their oncologic outcomes. Another multi-center analysis by Al Abbas et al. (2023, NSQIP database) [56] compared 476 MIPD vs. 4907 OPD after neoadjuvant chemo. MIPD in this setting was found to be safe and possibly advantageous: the MIPD group had lower overall complication rates, shorter hospitalization, and fewer clinically relevant pancreatic fistulas (CR-POPF). In fact, on multivariable analysis, MIPD was an independent predictor of reduced CR-POPF (OR 0.58, 95% CI 0.35–0.96, *p* = 0.04). This somewhat surprising finding of fewer fistulas after NAT followed by MIPD should be interpreted with caution, as another RCT, which did not focus specifically on NAT, found no difference in fistula rates between minimally invasive and open surgery. It is possible that patients who made it to MIPD were a subset with favorable anatomy or good response to NAT, thereby introducing selection bias.

Looking ahead, a prospective evaluation is underway. The ongoing CSPAC-5 trial in China is a randomized study explicitly comparing laparoscopic vs. open PD in patients with borderline resectable PDAC after neoadjuvant therapy [57]. Its results will provide high-level evidence on whether MIPD can be standard in the NAT context. For now, the pattern is that experienced centers are increasingly comfortable performing MIPD after NAT, with promising early outcomes such as the absence of excess mortality or positive margins [31,32]. Surgeons attempting this must be prepared for dense desmoplastic reaction—advanced robotic systems and use of techniques like artery-first approaches can help navigate fibrotic planes. In summary, NAT is no longer an absolute barrier to MIPD; with multidisciplinary planning and selective patient criteria, minimally invasive resection can be extended to these borderline cases, potentially allowing patients to reap MIS benefits even after intense preoperative therapy.

### 5.2. Frail and Elderly Patients

Patients with pancreatic cancer are disproportionately older, and a substantial proportion present with significant comorbidities or clinical frailty. “Frailty” refers to a state of decreased physiologic reserve and increased vulnerability to stressors, common in the elderly or chronically ill [58]. About 10% of adults over 65 are estimated to be frail, and frailty is associated with higher risks of postoperative complications and failure to rescue [59]. Intuitively, one might think frail patients should not undergo a complex operation like PD at all. But when surgery is pursued, the question arises: is a minimally invasive approach better or worse for a frail patient compared to open surgery? On one hand, MIS could reduce the surgical stress, which might benefit frail patients. On the other hand, longer operative times and risk of complications could be more dangerous for them.

Evidence, though limited, suggests that frail patients may indeed fare better with MIPD versus OPD, if they are carefully selected. Farah et al. performed a large retrospective analysis using the pancreatectomy-targeted ACS NSQIP data (2014–2021) focusing on frail patients (defined by a modified frailty index) who underwent PD [59]. Out of 3143 frail patients, only 9% had MIPD—again reflecting that MIS was only offered to a subset. Importantly, after adjusting for other factors, MIPD was associated with significantly better outcomes in frail patients: lower overall complications (43% vs. 54% with open, *p* < 0.001), lower major complication rate (29% vs. 35%, *p* = 0.04), and fewer discharges to nursing or rehab facilities (12% vs. 17%, *p* = 0.02). Additionally, within the MIPD group, robotic surgery seemed superior to straight laparoscopy for frail patients—robotic cases had fewer complications (39% vs. 51%) and lower 30-day mortality (1% vs. 4%) than laparoscopic cases. These findings suggest that, when feasible, a minimally invasive approach—particularly the robotic platform with its ergonomic and visual advantages—can mitigate surgical trauma in vulnerable patients, thereby translating to a tangible reduction in morbidity. Another meta-analysis which likely pooled various studies of elderly/frail subsets, reported a significantly lower 90-day mortality for MIPD vs. OPD (OR 0.56) and lower incidence of delayed gastric emptying (OR 0.54) [60]. Other outcomes like fistula, bleeding, or reoperation did not differ significantly—meaning MIS did not incur higher risk in those categories.

However, interpreting these results must acknowledge selection bias: surgeons likely chose frail patients for MIPD only when they believed the patient could tolerate it and when the tumor was anatomically favorable. Frail patients with very advanced disease or high complexity may have all been funneled to open surgery or deemed inoperable, leaving a relatively “healthier frail” group for MIS. This could partially explain the better outcomes observed. Nevertheless, it is plausible that reduced wound complications, less analgesic requirement, and quicker mobilization with MIS have disproportionate benefits in frail individuals who cannot afford setbacks in recovery. If a complication does occur, though, frail patients are at high risk regardless of approach, so careful preoperative optimization and geriatric assessment are key.

In summary, MIPD appears to be a favorable approach for well-selected frail or elderly patients, offering them a gentler recovery and lower risk of institutionalization post-surgery [60,61,62,63]. The caveat is that such patients should be managed in centers capable of high-level perioperative care, and perhaps with geriatric co-management. As the population ages, recognizing and measuring frailty should become part of PD patient workup. Those identified as frail might benefit from an MIS approach if feasible—but if a center does not have MIS expertise, forcing a frail patient through a surgeon’s MIPD learning curve would be counterproductive. Therefore, expanding MIS to frail patients should go hand-in-hand with ensuring the procedure is performed by experienced teams. Looking forward, increased access to MIPD could extend its benefits to this vulnerable group, potentially improving their postoperative quality of life and independence.

### 5.3. Tumors Requiring Vascular Resection

Involvement of the mesenteric vasculature (portal vein, superior mesenteric vein or artery) by tumor has traditionally been considered a relative contraindication to MIPD [64]. Such cases require vascular resection and reconstruction, adding complexity and potential bleeding risk. Historically, surgeons felt more secure performing these under open exposure [65]. However, top centers have begun performing MIPD with concurrent vascular resection (MIPD-VR), particularly using the robotic platform which offers fine control for vascular suturing [66,67,68]. The question is whether outcomes are comparable to open vascular resection, and early data suggests they can be, albeit with longer operative times.

Recent series from high-volume centers have reported that minimally invasive pancreatoduodenectomy combined with concurrent vascular resection can be performed safely [69,70]. For example, Napoli et al. demonstrated that robotic pancreatoduodenectomy with vein resection was non-inferior to open pancreatoduodenectomy with vein resection in a propensity-matched cohort, with comparable severe complication rates and similar oncologic outcomes [69]. Other groups have also reported feasibility of laparoscopic approaches with portal vein/superior mesenteric vein resection and reconstruction [66]. Importantly, they were able to perform a variety of vein reconstructions (wedge resection with primary repair, segmental resection with end-to-end anastomosis, and graft interposition) using MIS techniques. This demonstrates the technical versatility now achievable with robotics—maneuvers once thought only possible via open surgery can be performed with similar efficacy through small incisions by highly skilled teams.

A systematic review and meta-analysis by Mejía et al. (2024) pooled outcomes of robotic PD with portal-mesenteric vein resection (RPD+VR) versus open (OPD+VR) [66]. Across 4 studies with 288 patients (69 robotic vs. 219 open), they found no statistically significant differences in key outcomes: 30-day mortality, major complications, clinically relevant fistula, DGE, and post-pancreatectomy hemorrhage rates were all similar between RPD+VR and open. The one difference was that robotic cases had a significantly shorter hospital stay by 4.76 days on average. This aligns with the general trend that the minimally invasive approach can reduce hospital stay even when major adjunct procedures like vein resection are involved. The authors concluded that robotic PD with vein resection is a safe and effective alternative to open, conferring the usual recovery benefits without sacrificing oncologic or surgical safety.

It should be emphasized that these results come from specialized centers already adept in both MIPD and vascular surgery. Case selection remains critical—for instance, tumors requiring very complex arterial resections or multi-vessel involvement are still usually performed open. Robotic technology improvements may further ease vascular resections minimally invasively. At present, vascular resection during MIPD is an emerging but feasible frontier, with mounting evidence that it can be performed without compromise in outcomes. Patients with borderline resectable tumors who were once uniformly assigned to open surgery might now have the option of a minimally invasive resection in experienced hands.

Beyond the scenarios discussed above, surgeons have reported favorable outcomes in areas previously considered contraindications for MIS, such as large tumors exceeding 4–5 cm [6,70]. Traditionally, a tumor diameter greater than 3–4 cm was a relative contraindication for MIPD due to concerns regarding exposure and oncologic handling. However, recent series describe the application of MIPD for larger tumors. Some surgeons employ a laparoscopic “in situ no-touch isolation” technique, in which the tumor is devascularized and isolated prior to manipulation. Shen et al. specifically demonstrated this no-touch laparoscopic technique for PDAC, aiming to adhere to oncologic principles while minimizing tumor handling [70].

The feasibility of such specialized approaches suggests that tumor size alone may not constitute an absolute contraindication, provided that oncologic principles—such as avoidance of tumor rupture and achievement of negative margins—are strictly adhered to. However, the available evidence is derived largely from highly selected cases and remains limited, raising concerns regarding selection bias. Accordingly, very large tumors, particularly those obscuring critical vascular or anatomic landmarks, should still be approached with open surgery unless managed at experienced, high-volume minimally invasive centers [71].

Regarding redo or palliative surgery, there is limited but noteworthy exploration of MIPD in complex settings, such as remnant pancreatic cancer resection or palliative double bypass procedures [72,73,74]. Although these scenarios are not the primary focus of this review, the technical principles established in standard MIPD are increasingly being applied to these niche indications with appropriate caution.

In summary, the indication spectrum for MIPD is broadening. Where once only low-risk cases were attempted, now cases with prior therapy, frailty, and even vascular involvement are being performed minimally invasively with encouraging results. Each expansion is supported by at least some evidence that outcomes are comparable to open surgery in expert hands. Still, these are not interventions to be undertaken lightly; careful patient selection, informed consent and a readiness to convert to open if needed remain paramount. This expansion also reinforces the earlier points about learning curve and centralization—these complex MIS cases should be concentrated in centers that have demonstrated excellence in simpler MIPD cases first.

## 6. Cost and Resource Utilization

Beyond technical considerations, MIPD also carries important economic and resource implications. Robotic platforms in particular require substantial capital investment, ongoing maintenance, and disposable instrument costs, while longer operative times increase operating room resource utilization [33,75]. Although shorter hospital stays and reduced transfusion requirements may partially offset these expenses in high-performing centers, current cost-effectiveness analyses remain mixed and appear highly sensitive to institutional efficiency and complication rates [76]. It should be noted that cost-effectiveness is highly context-dependent—analyses may reach different conclusions in publicly funded healthcare systems versus private or mixed-payor systems. Furthermore, access to advanced platforms and training infrastructure varies widely across healthcare systems, limiting the global dissemination of MIPD and concentrating its use in specialized centers. These factors suggest that adoption of MIPD should be aligned with institutional capacity and supported by transparent reporting of both clinical and economic outcomes, while future studies incorporating quality-of-life and downstream cost metrics will be essential to better define its value in pancreatic cancer care.

## 7. Conclusions

In conclusion, MIPD represents a significant advance in the surgical management of pancreatic cancer, embodying the progress of surgical innovation aimed at reducing patient trauma while maintaining oncologic rigor. The current evidence supports MIPD as a valid alternative to open surgery in appropriately selected patients treated by experienced surgeons. To fully integrate MIPD into standard pancreatic cancer care, the surgical community must continue to pursue high-quality evidence, embrace new technologies responsibly, and ensure that the dissemination of MIPD does not outpace the ability to do it well. With ongoing commitment to research, education, and patient-centered outcomes, the coming years should clarify MIPD’s ultimate impact—determining whether it can truly deliver improved recovery without compromising oncologic outcomes in the battle against one of the most formidable cancers.

## Figures and Tables

**Table 1:** Key randomized trials comparing minimally invasive (laparoscopic or robotic) versus open pancreatoduodenectomy. LOS = length of hospital stay; R0 = microscopically margin-negative resection; LN = lymph node; DGE = delayed gastric emptying; CCI = Comprehensive Complication Index.

Trial (Year)	Patients (MIPD vs. OPD)	Tumor Types	Key Endpoints	Key Findings
Palanivelu et al., 2017 [29]	64 (LPD 32 vs. OPD 32)	Periampullary (Single center, India; pancreatic cancer, 17.2%)	Primary: LOS; Secondary: blood loss, R0 margin, LNs, complications	LPD had shorter median LOS (7 vs. 13 days, *p* < 0.001) and less blood loss (250 vs. 401 mL, *p* < 0.001). Operative time was longer for LPD. No differences in R0 resection, lymph nodes harvested, or major complications; mortality 3% in each arm.
Poves et al. (PADULAP), 2018 [30]	66 (LPD 34 vs. OPD 32)	Periampullary (Single center, Spain; pancreatic cancer, 59%)	Primary: LOS; Secondary: operative time, Clavien ≥ 3 complications, CCI score, “poor quality outcome”, margin/LNs	LPD had shorter LOS (median 13.5 vs. 17 days, *p* = 0.024). LPD had longer OR time (486 vs. 365 min). Severe complications were fewer with LPD (5 vs. 11 patients, *p* < 0.05); comprehensive complication index and composite poor-outcome measures favored LPD. No differences in transfusions, pancreatic fistula, DGE, R0 rate or LN yield.
van Hilst et al. (LEOPARD-2), 2019 [27]	99 (LPD 50 vs. OPD 49)	Periampullary (Multicenter, Netherlands; pancreatic cancer, 29.3%)	Primary: functional recovery; Secondary: Serious complication rate	Trial stopped early due to safety: 90-day mortality was higher in LPD group (approx. 8% vs. 2% in OPD) leading to termination. No significant differences in overall complication rates were observed before stopping, but concern for learning-curve effect was noted. Emphasized importance of surgeon experience and caution in new adopters.
Wang et al., 2021 [31]	594 (LPD 297 vs. OPD 297)	Periampullary (multicenter, China pancreatic cancer, 33.8%)	Primary: LOS; Secondary: estimated intraoperative blood loss, operative time, complication rate, mortality, CCI	LPD by expert surgeons showed lower overall complications (49% vs. 71%, *p* < 0.01) and fewer severe complications (12% vs. 22%). Postoperative LOS was modestly shorter (by 1 day). No differences in 90-day mortality or oncologic outcomes (R0 resection, LN yield). Concluded LPD is feasible and safe in high-volume settings.
Yoon et al. (K-MIPS), 2024 [32]	252 (LPD 125 vs. OPD 127)	Periampullary (multicenter, Korea; pancreatic cancer, 23.1%)	Primary: Functional recovery Secondary: complications, oncologic metrics, time to adjuvant therapy	LPD significantly improved functional recovery time (median 7.7 vs. 9.0 days, *p* = 0.03). No differences in major complications, 90-day mortality, R0 resection rate or lymph node count. Demonstrated holistic recovery benefits with MIPD when performed by experienced surgeons, while maintaining equivalent oncologic and safety outcomes.
Klotz et al. (EUROPA, robotic), 2024 [33]	62 (RPD 29 vs. OPD 33)	Periampullary (single center, Germany; pancreatic cancer, 35.48%)	Primary: cumulative morbidity within 90 days after PD; Secondary: CCI, CR-POPF, costs, conversion rate	No difference in overall morbidity (mean CCI 34.02 vs. 36.45, *p* = 0.71). RPD had more CR-POPF (58.6% vs. 33.3%, *p* = 0.046), longer operative time, and higher median costs. Conversion to open was required in 23% of RPD cases. 90-day mortality was <5% overall (acceptable for both arms). Concluded that in an expert center RPD is feasible but not superior to OPD, and highlighted the resource and cost drawbacks.
Liu et al. (China), 2024 [34]	161 (RPD 81 vs. OPD 80)	Periampullary (multicenter, China; pancreatic cancer, 35.4%)	Primary: LOS; Secondary: operative time, complications	RPD achieved shorter median hospital stay (11.0 vs. 13.5 days, *p* = 0.03). No significant differences in major complication rates (Clavien ≥ 3) or 90-day mortality (1% in each arm). R0 resection and lymph node yield were comparable (13 nodes in each). Notably, adjuvant therapy was initiated sooner after RPD (by 6.5 days on average). Demonstrated that RPD can enhance recovery without compromising safety or oncologic efficacy in experienced hands.

**Table 2:** Oncologic Outcomes of MIPD versus OPD in Pancreatic Cancer.

Study (Year)	Study Design	Patients (MIPD vs. OPD)	MIPD Type	R0 Resection Rate	Lymph Nodes Retrieved	Disease-Free Survival (DFS)	Overall Survival (OS)	Adjuvant Therapy	Key Findings
Croome et al., 2014 [11]	Retrospective, single-center (USA)	LPD, 108 vs. OPD, 214	LPD	No difference in margin status (LPD 77.8% vs. OPD 76.6%)	Not specifically reported	Longer progression-free survival in LPD group	No difference in OS (*p* = 0.12)	LPD patients had significantly lower proportion with >90-day delay or omission of adjuvant therapy: 5% vs. 12% in OPD (*p* = 0.04)	Faster recovery with LPD corresponded with longer progression-free survival. Earlier initiation of adjuvant chemotherapy may confer survival benefit.
Nussbaum et al., 2016 [39]	Retrospective, National Cancer Data Base (USA)	MIPD, 1191 vs. OPD, 6776	LPD & RPD	no difference in the rate of positive margins (20.4 vs. 22.1%; *p* = 0.15)	MIPD, 17.4 vs. OPD 16.5; *p* = 0.01	Not reported	2-year OS: equivalent	Found no significant improvement in adjuvant therapy use or timing with MIPD for PDAC (OR 1.00; *p* = 0.98).	Large database analysis suggesting that in broader practice, the advantage of earlier adjuvant therapy may not consistently manifest with MIPD.
Jiang et al., 2019 [40]	Meta-analysis (8 studies)	>15,000 PDAC patients total	LPD	LPD achieved higher R0 rate than OPD	LPD harvested more lymph nodes than OPD	Not separately reported	5-year OS: equivalent	Not reported	Meta-analysis specifically focused on PDAC. LPD showed slightly better odds of negative margins and greater number of nodes examined, likely reflecting meticulous dissection and enhanced visualization. Long-term survival equivalent.
Choi et al., 2020 [9]	Retrospective, single-center (Korea)	LPD, 27 vs. OPD 34	LPD	No difference in margin status	Not specifically reported	LPD, 34.2 vs. OPD, 23.3 months, *p* < 0.05	LPD, 44.6 vs. OPD, 45.3 months, *p* = 0.223	Found no significant improvement in adjuvant therapy	Improved disease-free survival for LPD over OPD in PDAC, though overall survival was similar. Suggests MIPD does not compromise curative intent.
Da Dong et al., 2021 [41]	Meta-analysis	RPD vs. OPD	RPD	RPD provides better histopathological outcomes compared to open	RPD associated with improved lymph node harvest (MD, 2.88 (1.12, 4.65); *p* = 0.001	Not reported	Not reported	Not reported	Meta-analysis showing robotic approach provides better histopathological outcomes. Improved visualization and fine instrument control facilitate thorough lymphadenectomy.
Wang et al., 2023 [42]	RCT, multicenter (China)	LPD, 100 vs. OPD 100	LPD	Not reported in short-term analysis	No difference in positive resection margin (*p* = 0.25)	Not yet reported (short-term outcomes only)	Not yet reported (short-term outcomes only)	Not reported	RCT specifically in PDAC patients. Showed no difference in 30-day morbidity or mortality between approaches. Confirmed expected longer operative time and lower blood loss with LPD. Long-term follow-up needed for survival data.

MIPD = minimally invasive pancreatoduodenectomy; OPD = open pancreatoduodenectomy; LPD = laparoscopic pancreatoduodenectomy; RPD = robotic pancreatoduodenectomy; PDAC = pancreatic ductal adenocarcinoma; R0 = microscopically margin-negative resection; DFS = disease-free survival; OS = overall survival; RCT = randomized controlled trial.

## Data Availability

Data sharing is not applicable to this article. All sources of information analyzed during this study are included in the published article (and its reference list).
